# Aging-related decrements during specific phases of the dual-task Timed Up-and-Go test

**DOI:** 10.1007/s40520-015-0372-x

**Published:** 2015-05-21

**Authors:** Franchino S. Porciuncula, Ashwini K. Rao, Tara L. McIsaac

**Affiliations:** Department of Biobehavioral Sciences, Teachers College Columbia University, Movement Sciences, Box 199, 525 West 120th St., New York, NY 10027 USA; Department of Rehabilitation and Regenerative Medicine (Physical Therapy) and G.H. Sergievsky Center, College of Physicians and Surgeons, Columbia University, 710 West 168th St., 8th Floor, New York, NY 10032 USA; Department of Physical Therapy, Arizona School of Health Sciences, A.T. Still University, 5850 E. Still Circle, Mesa, AZ 85206 USA

**Keywords:** Dual-task, Gait, Attention, Cognition, Aging

## Abstract

**Background:**

It is unclear how young and older adults modulate dual-task mobility under changing postural challenges.

**Aim:**

To examine age-related changes in dual-task processing during specific phases of dual-task Timed Up-and-Go (TUG_dual-task_).

**Method:**

Healthy young and older adults performed the Timed Up-and-Go (TUG) with the following dual-task conditions: (1) serial-three subtractions, (2) carrying cup of water, (3) combined subtraction and carrying water, and (4) dialing cell phone. The primary outcome was the dual-task cost on performance of TUG (percent change from single- to dual-task) based on duration and peak trunk velocity of each phase: (a) straight-walk, (b) sit-to-stand, (c) turn, (d) turn-to-sit. Mixed-design univariate analysis of variance was performed for each type of task.

**Results:**

Older adults had more pronounced mobility decrements than young adults during straight-ahead walking and turns when the secondary task engaged both cognitive and manual modalities. Simple cognitive or manual tasks during TUG_dual-task_ did not differentiate young from older participants. Subtraction performance during simple and complex cognitive conditions differed by phase of the TUG. Manual task performance of carrying water did not vary by phase or age.

**Discussion:**

Our findings suggest that dual-task processing is dynamic across phases of TUG_dual-task_. Aging-related dual-task decrements are demonstrated during straight-ahead walking and turning, particularly when the secondary task is more complex.

**Conclusion:**

Older adults are susceptible to reduced dual-task mobility during straight-ahead walking and turning particularly when attentional loading was increased.

## Introduction

Gait in older adults is compromised during dual-task conditions [1, 2], such that speed and stride length are reduced, and stride time and its variability are increased [3, 4]. Dual-task-related gait decrements can lead to instability and increased fall risk [5]. Falls are the leading cause of accidental deaths among older adults [6], thus understanding mobility and fall risk may reduce this burden. Cognitive functions, particularly attention, are necessary during gait; hence, gait decrements during dual-tasks can be explained by limited capacity of attention processing, or due to competition for cognitive resources [7, 8].

Research on dual-task gait is largely based on studies examining straight-ahead walking, yet most daily activities require transition movements, such as turns and sit-to-stand. Mechanics of turning deteriorate with age [9] wherein a simplified turning pattern can predict recurrent falls in the elderly [10]. The instability during turns is likely due to the unique physiologic and cognitive requirements of turns relative to straight-ahead walking [11, 12]. For example, cognitive processing speed was found to be uniquely associated with curvilinear walking but not with straight-ahead walking [11]. It remains unclear how older adults manage dual-task mobility when the postural requirements of component tasks differ, as in linear and curved walking.

The Timed Up-and-Go test (TUG), a clinical test of mobility and fall risk in older adults, includes straight-ahead walking and transitions [13]. Dual-tasks have been integrated into the TUG (TUG_dual-task_) [2, 14]; however, these studies analyzed the total TUG duration rather than its individual phases, and thus may not be successful in assessing at-risk older adults. For instance, Shumway-Cook et al. [14] demonstrated that adding a dual-task challenge failed to enhance falls prediction more than that of the regular TUG. Although based on the conventional TUG, Mirelman et al. [15] demonstrated that the total TUG duration failed to differentiate older adults with and without cognitive impairment, but performance in specific phases did. Further, kinematic data during specific phases were found to be independent of the total duration [16]. No study thus far has examined phases of the TUG_dual-task_. Understanding dual-task behavior during individual phases may reveal subclinical mobility changes that may assist in early and targeted intervention.

The first objective was to examine age-related decrements in mobility during phases of the TUG_dual-task_. We hypothesized that older adults would demonstrate greater dual-task decrement of duration and peak velocity during transitions of the TUG compared to young adults. The second objective was to characterize dual-task performance during specific phases of the TUG when engaged in simple and complex secondary tasks. We hypothesized that dual-task decrements of duration and peak velocity will be greater during transitions and when secondary tasks are more complex.

## Methods

### Participants

Twelve healthy young adults (mean ± standard deviation, *M* ± SD: 26.13 ± 5.36 years) and 12 older adults (*M* ± SD: 74.18 ± 5.21 years) (see Table 1) were recruited from the community and university population. Physical therapists performed medical history taking, clinical screening of gross mobility, and vibration testing of the foot and ankle. Participants were included if they were able to independently ambulate in the community, follow instructions in English, and tolerate a 2-h testing session. Exclusion criteria were impaired vibration sense of feet or ankles, diagnosis of dementia, or any neurological, orthopedic, or medical condition that impaired walking. The Institutional Review Board approved this study, and participants provided signed informed consent.

**Table 1:** Comparison of characteristics between young and old

	Young (*n* = 12)	Old (*n* = 12)	*p* value^a^
	Mean ± SD	Mean ± SD	
Subject characteristics			
Height (cm)	166.58 ± 8.92	168.06 ± 8.92	0.689
Age (years)	26.13 ± 5.36	74.18 ± 5.21	–
Education (years)	16.67 ± 2.74	16.33 ± 5.77	0.858
MoCA	28.88 ± 1.13	26.55 ± 1.92	0.007*

* Significant

^a^ two-tailed significance from independent samples *t* test

*MoCA* Montreal Cognitive Assessment, *SD* Standard deviation

### Apparatus

Movements were recorded using six wireless inertial sensors (Opal ™, APDM, Portland, OR, USA), with dimensions of 48.4 × 36.1 × 13.4 mm. Sensors were secured to wrists and ankles bilaterally, and mid-thoracic and lower lumbar areas using Velcro straps. Trials were audio–video recorded for analysis of secondary tasks.

### Tasks and procedures

**Fig. 1:**
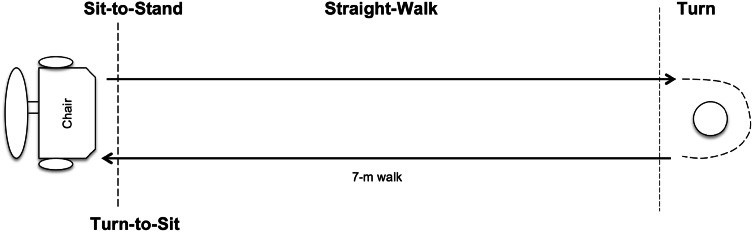
Phases of the instrumented Timed Up-and-Go

Mobility was assessed using the 7-m TUG, instrumented with inertial sensors (iTUG) (Fig. 1) [17]. The single-task iTUG was performed as follows: upon cue, the subject stood up from a chair without hand support (Sit-to-Stand), walked straight-ahead 7 m, turned around (Turn), walked back to the chair, turned and sat down (Turn-to-Sit). Periods of walking to and from the 7-m mark were consolidated into a single straight-ahead walk (Straight-Walk). The phases of interest were Straight-Walk, Sit-to-Stand, Turn, and Turn-to-Sit. The single-task iTUG served as reference for each participant’s dual-task performance. We selected four conditions for the TUG_dual-task_: (1) serial-subtraction by 3′s from a random number between 70 and 99 (COUNT); (2) carrying cup of water filled up to 1 cm below rim (CARRY); (3) combined COUNT and CARRY (CtCARRY); and (4) dialing home phone number with cell phone (DIAL). The DIAL condition served as alternative to CtCARRY, examining the effect of combined cognitive–manual processing, without structural interference from water dynamics seen in CtCARRY. Because integration of cognitive and manual tasks was required during CtCARRY and DIAL, these were considered complex tasks, while COUNT and CARRY were considered simple tasks. Subjects were instructed to walk as quickly as possible, and perform the secondary task as quickly and/or accurately as possible. Each subject performed three trials per condition in pseudo-randomized order.

### Analysis

#### Outcome measures

Our outcome measures were phase-specific duration and peak velocities of the iTUG_dual-task_, because these measures were available for each phase, thus allowing comparison of similar mobility constructs [18] across phases. Measures were obtained through Mobility Lab™ software (APDM, Eugene, OR) utilizing algorithms by Salarian et al. [17, 19]. Duration (s) refers to the time to complete each phase, while peak velocity (°/s) refers to 95 % of peak angular velocity of trunk per individual phase.

Next, we calculated the dual-task cost (DTC), defined as the percent change in performance relative to an individual’s single-task performance [20]. This normalized the data and distinguished dual-task processing from usual age-related changes. The DTC was computed for duration (Eq. 1) and peak velocity (Eq. 2), with negative and positive multipliers used, respectively, for directionality of performance decrement.

1 $$ \% {\text{DTC }} = - [ \, ({\text{Dual}} - {\text{task}} - {\text{Single}} - {\text{task}}) \, / \, \left( {{\text{Single}} - {\text{task}}} \right) \, ] \, \times \, 100 $$

2 $$ \% {\text{DTC }} = \, + \, [ \, ({\text{Dual}} - {\text{task}} - {\text{Single}} - {\text{task}}) \, / \, \left( {{\text{Single}} - {\text{task}}} \right) \, ] \, \times \, 100 $$

The greater DTC value in the negative direction implied greater performance decrements.

To assess manual performance, we recorded the number of spills per phase during simple (CARRY) and complex (CtCARRY) manual conditions. To assess cognitive performance, we examined response rate (Eq. 3), and response accuracy (Eq. 4) during simple (COUNT) and complex (CtCARRY) cognitive conditions. Both outcomes were expressed in percent for simplicity of interpretation.

3 $$ \% {\text{Response rate }} = \, \left( {\# {\text{responses per phase}}/{\text{phase duration}}} \right) \times 100 $$

4 $$ \% {\text{Response accuracy }} = \, \left( {\# {\text{responses per phase }}{-} \, \# {\text{ errors per phase}}} \right)/\left( {\# {\text{responses per phase}}} \right) \times 100 $$

Recent studies [20–22] examined both single- and dual-task performance of the secondary tasks to assess interplay of gait and secondary tasks. Our study did not adopt this methodology because we were interested in phase-specific performance rather than DTC of the entire TUG, and this methodology may not necessarily assist in answering questions related to phase-specific performance. Instead, we compared phase-specific change in performance of the secondary tasks between simple and complex conditions.

#### Statistical analysis

SPSS (Version 22) was used. Group means are reported as *M* and its 95 % confidence interval (CI). To determine the effect of age (Young, Old) and phase (Straight-Walk, Sit-to-Stand, Turn, Turn-to-Sit) on DTC of mobility measures, linear mixed models were utilized on duration and peak velocity per phase. We used a mixed-design univariate analysis of variance (ANOVA) with random-nested factor of subject, and fixed factors of age, group and phase. To examine differences in phases, post hoc pairwise comparisons were performed using Bonferroni corrections. Separate analyses were performed for each secondary task. Similar univariate ANOVA, as described above, was used to examine manual and cognitive performance, with analysis performed separately for each outcome per condition.

## Results

### Subjects

As summarized in Table 1, our sample of young (*N* = 12) and older adults (*N* = 12) did not differ in education, *t*_22_ = 0.181, *p* = 0.858, or height *t*_22_ = −0.405, *p* = 0.689. However, older adults had lower Montreal Cognitive Assessment (MoCA) scores than young adults, *t*_19_ = 3.06, *p* < 0.01. The average score of older adults (*M* = 26.55, SD = 1.92) was higher than reference values for mild cognitive impairment (*M* = 22.1, SD = 3.1; cutoff: ≤25) [23].

### TUG measures

#### Effect of phase on duration and peak velocity

**Fig. 2:**
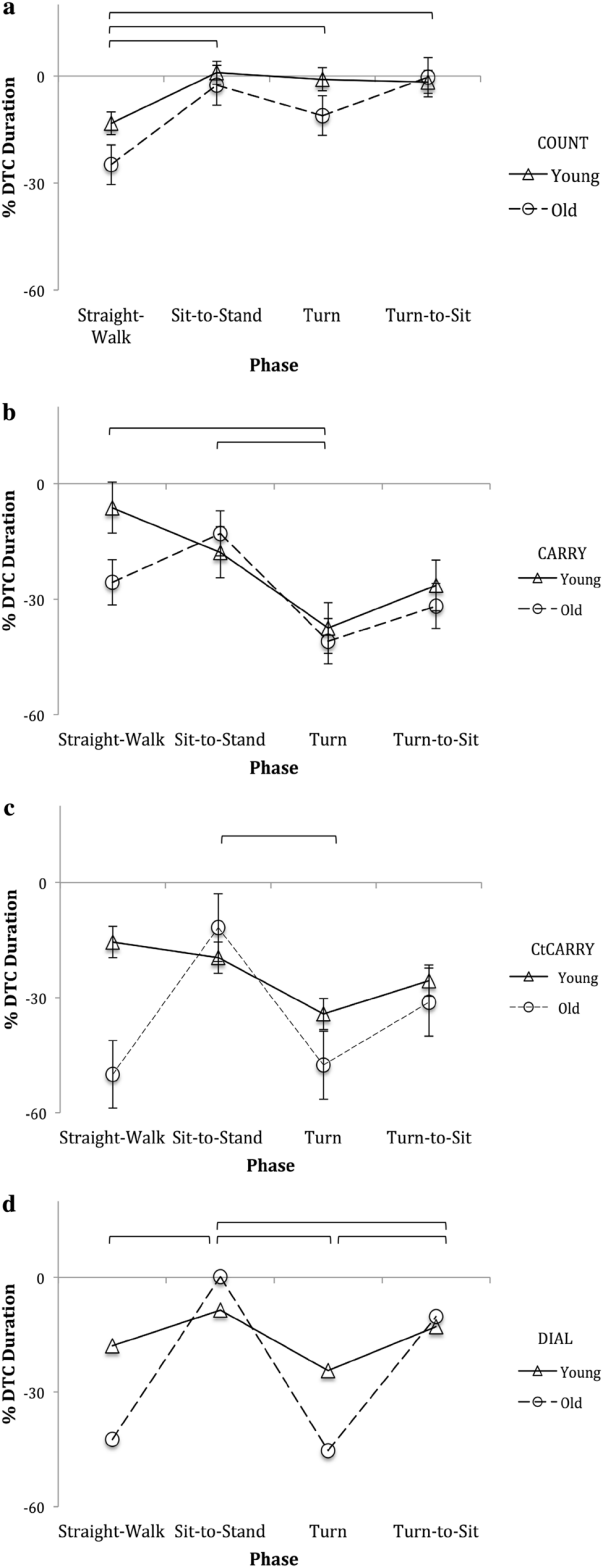
DTC on duration of young and old during TUG phases according to **a** COUNT, **b** CARRY, **c** CtCARRY, and **d** DIAL conditions. *DTC* Dual-task cost

**Fig. 3:**
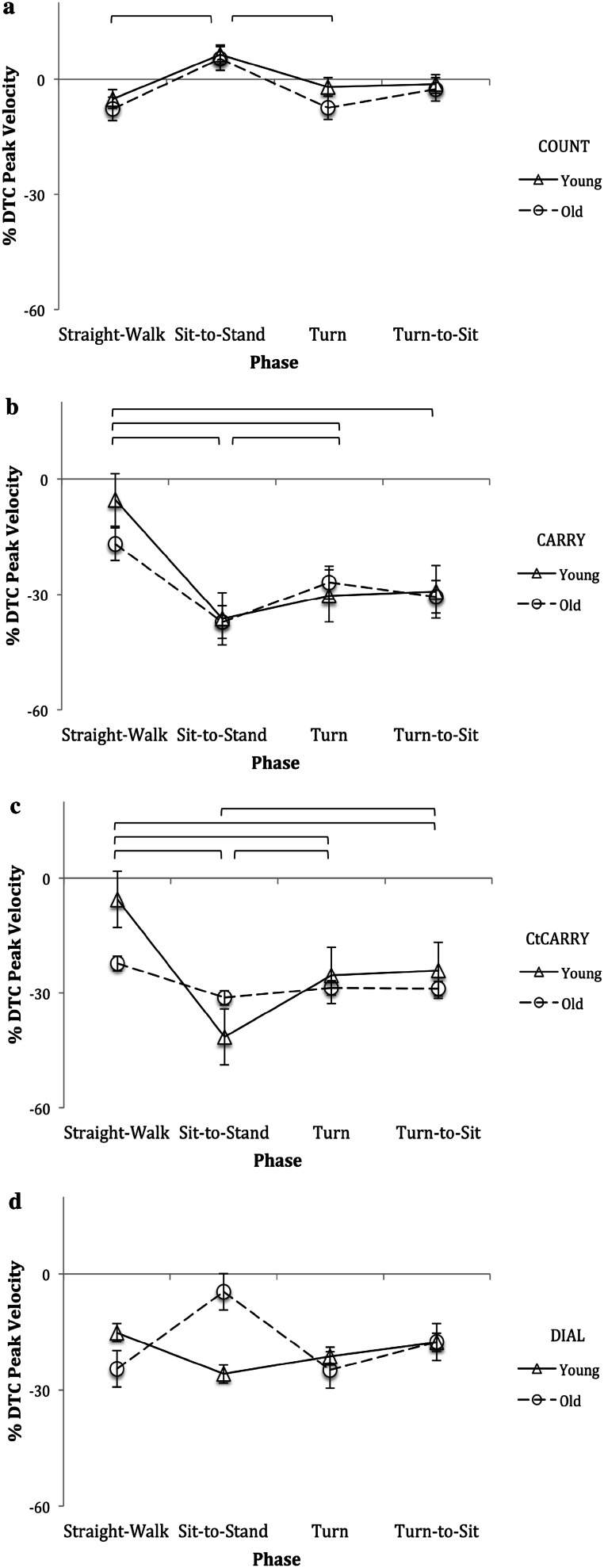
DTC on peak velocity of young and old during TUG phases according to **a** COUNT, **b** CARRY, **c** CtCARRY, and **d** DIAL conditions. *DTC* Dual-task cost, *TUG* Timed Up-and-Go, *COUNT* Cognitive task, *CARRY* Manual task, *CtCARRY* Cognitive–manual task, *DIAL* Phone task

The DTC on duration differed across phases of the iTUG depending on the dual-task condition, with main effect of phase in all conditions: COUNT (*F*(3, 66) = 9.021, *p* < 0.001), CARRY (*F*(3, 66) = 8.960, *p* < 0.001), CtCARRY (*F*(3, 66) = 4.892, *p* = 0.004), and DIAL (*F*(3, 66) = 16.034, *p* < 0.001) (Table 2; Fig. 2). Similarly, there was a main effect of phase for the DTC on peak velocity during COUNT (*F*(3, 63) = 4.242, *p* = 0.009), CARRY (*F*(3, 63) = 30.059, *p* < 0.001), and CtCARRY (*F*(3, 63) = 15.400, *p* < 0.001), but not for DIAL (*F*(3, 63) = 0.908, *p* = 0.442) (Table 2; Fig. 3). Thus, the DTC on duration and peak velocity varied according to the phase of the iTUG across conditions, with the exception of DIAL for peak velocity.

**Table 2:** Means (*M*), 95 % confidence intervals (CI) with corresponding *p* value and partial eta-squared (*η*_p_^2^) of dual-task cost (DTC) on duration and peak velocity (Peak_vel) during cognitive (COUNT), manual (CARRY), cognitive–manual (CtCARRY), and phone (DIAL) conditions for young (Y) and old (O)

Condition, variable, and age	Phase								*p*-value	*η*_*p*_^2^
	Straight-Walk		Sit-to-Stand		Turn		Turn-to-Sit			
	*M*	CI	*M*	CI	*M*	CI	*M*	CI	(Phase main effect, Age main effect, Age by Phase interaction)	
COUNT										
%DTE_Duration										
Y	−14.31	[−22.65, −5.97]	1.10	[−7.24, 9.433]	−0.96	[−9.29, 7.39]	−1.64	[−9.98, 6.70]	(<0.001, 0.057, 0.429)	(0.291, 0.054, 0.041)
O	−24.90	[−33.24, −16.57]	−2.60	[−10.94, 5.74]	−10.97	[−19.31, −2.63]	−0.18	[−8.51, 8.16]		
%DTE_Peak_vel										
Y	−6.01	[−13.88, 1.86]	5.60	[−2.27, 13.47]	−2.02	[−9.26, 5.22]	−2.07	[−9.94, 5.80]	(0.009, 0.472, 0.891)	(0.168, 0.008, 0.01)
O	−7.72	[−14.95, −0.48]	5.38	[−1.86, 12.61]	−7.45	[−14.69, −0.21]	−2.62	[−9.85, 4.62]		
CARRY										
%DTE_Duration										
Y	−6.44	[−17.22, 4.35]	−17.87	[−28.66, −7.08]	−37.45	[−48.24, −26.66]	−26.56	[−37.35, −15.77]	(<0.001, 0.139, 0.179)	(0.289, 0.033, 0.071)
O	−25.47	[−36.26, −14.68]	−12.98	[−23.76, −2.19]	−40.92	[−51.71, −30.14]	−31.83	[−42.62, −21.05]		
%DTE_Peak_vel										
Y	−7.61	[−13.55, −1.66]	−38.51	[−44.46, −32.57]	−30.34	[−35.81, −24.87]	−31.49	[−37.43, 25.54]	(<0.001, 0.655, 0.112)	(0.589, 0.003, 0.090)
O	−16.93	[−22.40, −11.46]	−37.19	[−42.66, −31.72]	−26.92	[−32.39, 21.45]	−30.61	[−36.07, −25.14]		
CtCARRY										
%DTE_Duration										
Y	−15.51	[−28.89, −2.124]	−19.60	[−32.98, −6.22]	−34.20	[−47.58, −20.81]	−25.60	[−38.98, −12.22]	(<0.004, 0.019, 0.021)	(0.182, 0.08, 0.136)
O	−49.86	[−63.24, −36.47]	−11.87	[−25.26, 1.51]	−47.51	[−60.89, −34.13]	−31.16	[−44.54, −17.78]		
%DTE_Peak_vel										
Y	−7.72	[−14.75, −0.70]	−43.63	[−50.65, −36.60]	−25.43	[−31.89, −18.97]	−26.26	[−33.28, −19.23]	(0.001, 0.410, 0.002)	(0.423, 0.011, 0.211)
O	−22.31	[−28.77, −15.85]	−31.24	[−37.70, −24.77]	−28.68	[−35.14, −22.22]	−28.90	[−35.63, −22.44]		
DIAL										
%DTE_Duration										
Y	−17.79	[−28.15, −7.42]	−8.50	[−18.87, 1.86]	−24.43	[−34.80, −14.07]	−12.88	[−23.24, −2.51]	(<0.001, 0.023, 0.003)	(0.422, 0.075, 0.191)
O	−42.44	[−52.81, −32.08]	0.208	[−10.16, 10.57]	−45.34	[−55.72, −34.99]	−10.07	[−20.44, 0.29]		
%DTE_Peak_vel										
Y	−17.72	[−26.62, −8.82]	−28.39	[−37.28, −19.49]	−21.27	[−29.45, −13.08]	−20.21	[−29.11, −11.31]	(0.442, 0.200, 0.002)	(0.041, 0.026, 0.204)
O	−24.54	[−32.73, −16.36]	−4.63	[−12.81, 3.56]	−24.82	[−33.00, −16.63]	−17.60	[−25.79, −9.42]

#### Effect of age on duration and peak velocity

There was an age-related difference of the DTC on duration only for the CtCARRY (*F*(1, 63) = 5.76, *p* = 0.019) and DIAL (*F*(1, 63) = 5.38, *p* = 0.023) conditions, but not for COUNT (*F*(1, 66) = 3.74, *p* = 0.057) and CARRY (*F*(1, 66) = 2.24, *p* = 0.139) (Table 1; Fig. 2). Conversely, when examining the DTC on peak velocity, there was no main effect of age under any condition: COUNT (*F*(1, 63) = 0.525, *p* = 0.472), CARRY (*F*(1, 63) = 0.201, *p* = 0.655), CtCARRY (*F*(1, 63) = 0.689, *p* = 0.410), and DIAL (*F*(1, 63) = 1.677, *p* = 0.200) (Table 2; Fig. 3). Thus, duration-related decrements were higher for older adults when secondary tasks were complex, but not during simple conditions. Peak velocity decrements were comparable between young and older adults.

#### Interaction between phase and age

There was a significant interaction between phase and age on DTC on duration during complex conditions, CtCARRY (*F*(3, 66) = 3.451, *p* = 0.021) and DIAL (*F*(3, 66) = 5.192, *p* = 0.003) (Fig. 2c, d), but not during simple conditions of COUNT (*F*(3, 66) = 0.936, *p* = 0.429) and CARRY (*F*(3, 66) = 1.685, *p* = 0.179) (Fig. 2a, b). Likewise, a significant interaction of age and phase on peak velocity was seen during complex conditions CtCARRY (*F*(3, 66) = 5.603, *p* = 0.002) and DIAL (*F*(3, 63) = 5.385, *p* = 0.002) (Fig. 3c, d), but not during simple COUNT (*F*(3, 63) = 0.208, *p* = 0.891) or CARRY (*F*(3, 63) = 2.079, *p* = 0.112) conditions (Fig. 3a, b).

Post hoc analysis revealed that dual-task decrement on duration was particularly evident for older adults during Straight-Walk and Turn phases during CtCARRY and DIAL (Fig. 2c, d), but not during COUNT and CARRY conditions (Fig. 2a, b). Furthermore, DTC on peak velocity during Straight-Walk was worse for older than younger adults during CtCARRY and DIAL (Fig. 3c, d). Taken together, this suggests that older adults dampened trunk velocities and extended duration during Straight-Walk, as well as lengthened Turn phases when secondary tasks required integration of cognitive and manual processing.

#### Cognitive performance

As summarized in Table 3, response rate differed depending on the phase of the iTUG for both COUNT (*F*(3, 57) = 45.136, *p* < 0.001) and CtCARRY (*F*(3, 57) = 63.647, *p* < 0.001). Furthermore, a significant main effect of age on response rate was found, where older adults had lower response rates than young adults in CtCARRY (*F*(1, 57) = 5.134, *p* = 0.027), but not during COUNT (*F*(1, 57) = 3.358, *p* = 0.072). There was no interaction between age and phase for either COUNT (*F*(3, 57) = 1.087, *p* = 0.362) or CtCARRY (*F*(3, 57) = 1.791, *p* = 0.159).

**Table 3:** Means (*M*), 95 % confidence intervals (CI) with corresponding *p* value and partial eta-squared (*η*_*p*_^2^) of counting performance during cognitive (COUNT) and cognitive–manual (CtCARRY) conditions for young (Y) and old (O)

Condition, variable, and age	Phase									
	Straight-Walk		Sit-to-Stand		Turn		Turn-to-Sit		*p*-value	*η*_*p*_^2^
	*M*	CI	*M*	CI	*M*	CI	*M*	CI	(Phase main effect, Age main effect, Age by Phase interaction)	
Simple (COUNT) Response rate (%)										
Y	81.90	[69.63, 94.16]	6.39	[−5.88, 18.65]	51.74	[39.47, 64.00]	29.21	[16.95, 41.48]	(<0.001, 0.072, 0.362)	(0.704, 0.056, 0.054)
O	67.05	[55.35, 78.74]	7.20	[−4.50, 18.89]	35.87	[24.18, 47.57]	28.10	[16.41, 39.80]		
Accuracy (%)										
Y	97.41	[76.06, 118.75]	30.00	[8.65, 51.35]	100.00	[78.65, 121.35]	83.33	[61.99, 104.68]	(<0.001, 0.104, 0.395)	(0.405, 0.046, 0.050)
O	87.05	[66.69, 107.40]	36.36	[16.01, 56.72]	70.46	[50.10, 90.81]	68.18	[47.83, 88.54]		
Complex (CtCARRY)										
Response rate (#/s)										
Y	82.06	[71.77, 92.35]	8.19	[−2.10, 18.49]	43.68	[33.39, 53.98]	25.69	[15.40, 35.98]	(<0.001, 0.027, 0.159)	(0.770, 0.083, 0.0.86)
O	69.16	[59.35, 78.98]	12.01	[2.20, 21.82]	25.73	[15.91, 35.54]	20.54	[10.73, 30.36]		
Accuracy (%)										
Y	91.23	[72.12, 110.34]	30.00	[10.89, 49.11]	94.67	[75.56, 113.78]	72.50	[53.39, 91.61]	(<0.001, 0.477, 0.321)	(0.387, 0.009, 0.059)
O	92.10	[73.88, 110.33]	54.55	[36.32, 72.77]	84.85	[66.63, 103.07]	75.76	[57.54, 93.98]		

*TUG* Timed Up-and-Go, *COUNT* Cognitive task, *CARRY* Manual task, *CtCARRY* Cognitive–manual task, *DIAL* Phone task

Accuracy differed by phase of the iTUG during COUNT (*F*(3, 57) = 12.933, *p* < 0.001), and CtCARRY (*F*(3, 57) = 12.009, *p* < 0.001). Age by itself did not affect the accuracy for either COUNT (*F*(1, 57) = 2.732, *p* = 0.104) or CtCARRY (*F*(1, 57) = 0.511, *p* = 0.477) conditions. There was no interaction between age and phase on subtraction accuracy on COUNT (*F*(3, 57) = 1.010, *p* = 0.395) or CtCARRY (*F*(3, 57) = 1.191, *p* = 0.321). Therefore, older adults had increased deficits in response rate during turns only when the task was complex. Accuracy of subtraction differed by phase, but did so similarly for both young and older adults.

#### Manual performance

Incidence of spills was not significantly different depending on phase, regardless of condition: CARRY (*F*(3, 66) = 2.033, *p* = 0.118; Straight-Walk: *M* = 0.097, CI (0.046, 0.148); Sit-to-Stand: *M* = 0.028, CI (−0.023, 0.079); Turn: *M* = 0.014, CI (−0.037, 0.065); Turn-to-Sit: *M* = *0*.042, CI (−0.009, 0.093)); CtCARRY (*F*(3, 66) = 1.903, *p* = 0.138; Straight-Walk: M = 0.069, CI (0.025, 0.114); Sit-to-Stand: *M* = 1.38 × 10^−17^, CI (−0.045, 0.045); Turn: *M* = 0.014, CI (−0.031, 0.058); Turn-to-Sit: *M* = 0.014; CI (−0.031, 0.058)). Age by itself did not affect the incidence of spills per phase during COUNT (*F*(1, 66) = 0.661, *p* = 0.419) and CtCARRY (*F*(1, 66) = 0.871, *p* = 0.354). No significant interaction was demonstrated between age and phase in CARRY (*F(*3, 66) = 0.073, *p* = 0.974) or CtCARRY (*F*(3, 66) = 0.097, *p* = 0.962). Therefore, both young and older adults consistently carried a cup of water throughout phases regardless of complexity of condition.

## Discussion

To our knowledge, this is the first study that investigated aging-related dual-task behavior during specific phases of the iTUG. Our results demonstrated pronounced aging-related decrements impacting duration of Straight-Walk and Turn phases, and peak velocity during Straight-Walk, particularly during complex conditions requiring cognitive–manual integration. These findings suggest that attentional processing is different across phases of an activity.

### Dual-task processing depends on phase of iTUG

Previous studies have shown that dual-task performance declines when postural challenge increases. Attentional demands were shown to progressively increase from sitting, standing, to walking [24]; or from standing to stair negotiation [25]. These studies, however, examined dual-task performance under separate tasks of varying postural challenge. Notably in our study, dual-task performance was assessed based on a sequence of tasks with different postural challenges, a scenario consistent with daily activity. Our findings suggest that attention processing is not at steady state throughout an activity; rather, it is different across phases of an activity.

Consistent with our hypothesis, older adults had pronounced decrements in duration during Turn particularly during complex conditions; however, we also find pronounced decrements in Straight-Walk. The high dual-task deficit in Straight-Walk seems less intuitive because straight-path walking is a well-learned task, thus should not highly tax attention resources. This may be explained by task prioritization. Straight-Walk may be regarded as low threat to stability relative to other transition phases. Thus, older adults may have diverted attentional resources to the secondary task at the expense of walking just because they can afford to do so [26]. Although we did not examine prioritization tradeoff, our results show that cognitive performance in COUNT and CtCARRY was improved during Straight-Walk relative to other phases, but at the expense of gait. Similarly, Patel et al. [22] demonstrated improved cognitive performance in young adults but at the expense of gait speed.

Age-related changes in grasp control can make Straight-Walk more complex. Diermayr et al. [27] demonstrated that coordination of grasp forces was compromised in older adults during challenged gait (obstacles) but not during regular walking, suggesting resultant deficits when other factors such as balance and attention were challenged. Similarly, in the current study, while not a grasp experiment, older adults had pronounced decrements in duration and peak velocity during Straight-Walk when conditions were complex (CtCARRY, DIAL). Manual performance across phases did not differ per group or condition (CARRY, CtCARRY), suggesting that dual-tasking mostly impacted gait and not the manual task. The findings of Diermayr et al. [27] along with our results suggest that modulation of gait and grasp control could be challenging even during straight-ahead walking when attentional loading is increased.

The longer time interval for Straight-Walk phase may be another factor for worse DTC. Straight-Walk, by design, was of longer duration than other phases in the TUG, and thus greater time may have provided more opportunity for errors or corrective responses (as in the subtraction task). It is possible that the DTC is time-sensitive, such that a more comprehensive assessment can be made when observations occur over a longer time interval.

### Dual-task processing depends on secondary task

Earlier reports have suggested that the type of task does not uniquely determine the extent of DTC [28]. For instance, previous studies demonstrated that cognitive (serial subtractions) and manual (coin transfer) secondary tasks rendered similar effects on gait [28, 29]. Our findings, however, revealed differentiation, such that CARRY generally caused intermediate DTC between COUNT and the combined tasks (CtCARRY, DIAL). Our study further expanded the conditions by integrating cognitive and manual modalities, allowing use of a broader range of secondary tasks common in daily activities. The greatest dual-task costs to the TUG occurred during CtCARRY and DIAL conditions, both of which comprised manual and cognitive components. Thus, the minimal to moderate DTC during COUNT or CARRY became additive when the secondary task involved both cognitive and manual components.

A significant interaction of age and phase on Straight-Walk duration was found in CtCARRY and DIAL. However, peak velocity had a significant interaction only for CtCARRY, but not for DIAL, likely due to differences in structural interference (water versus rigid object such as a phone). Overall, the greatest dual-task cost to iTUG was seen during more complex tasks, suggesting that type and complexity of secondary tasks matter in their effect on walking.

### Secondary task performance

Cognitive tasks may impact gait, just as gait may perturb cognitive performance [1]. We found that subtraction rate and accuracy was greatest during Straight-Walk, and was most impaired during Sit-to-Stand regardless of complexity of the secondary task (COUNT, CtCARRY). Overall, cognitive performance was similar across age groups. Hall et al. [30] demonstrated that cognitive factors could explain dual-task walking performance only when the cognitive challenges were sufficiently complex. Our older subjects had lower MoCA scores compared to young; nevertheless, both performed similarly in the subtraction task. Therefore, the cognitive task may not have been complex enough to load the cognitive systems in older adults. What may differentiate young and older adults in this study is dual-task performance during the iTUG.

### Limitations

Performance during the dialing task was not assessed due to technology limitations. This study examined a relatively small sample size; therefore, findings need to be interpreted cautiously.

## Conclusion

Attentional processing is different across phases of complex functional activities like the TUG. Older adults are more susceptible to dual-task mobility decrements during straight-ahead walking and turning, particularly when secondary tasks require integration of cognitive and manual modalities. Examination of these phases during clinical testing may assist clinicians in identifying at-risk individuals.
